# HDL dysfunction, function, and heart failure

**DOI:** 10.18632/aging.101775

**Published:** 2019-01-17

**Authors:** Mudit Mishra, Ilayaraja Muthuramu, Bart De Geest

**Affiliations:** 1 Centre for Molecular and Vascular Biology, Department of Cardiovascular Sciences, Catholic University of Leuven, Leuven, Belgium

**Keywords:** heart failure, HDL function, reconstituted HDL, apolipoprotein A-I gene therapy, scavenger receptor class B

Heart failure is the cardiovascular epidemic of the 21st century. One in five men and women will develop heart failure in their lifetime. The prevalence of heart failure can be estimated at 2% in the Western world and the incidence approaches 5–10 per 1000 persons per year. The prevalence of heart failure is 7% in the age group 75–84 years and over 10% in those older than 85 years. The 5-year age-adjusted mortality rates after onset of heart failure are 50% in men and 46% in women. Inhibition of the renin-angiotensin-aldosterone system and of the sympathetic nervous system both improve survival and decrease hospitalizations in patients with heart failure with reduced ejection fraction (HFrEF) (ejection fraction ≤40%). In contrast to these prominent advances in the treatment of HFrEF, drug strategies with strong evidence in HFrEF have proved unsuccessful in heart failure with preserved ejection fraction (HFpEF) (ejection fraction ≥50%) and mortality in HFpEF patients has remained unchanged. As the population ages, HFpEF will continue to be a growing public health problem [1,2]. HFpEF represents a major unmet therapeutic need.

High-density lipoproteins (HDL) consist of various subclasses, which share the abundance of apolipoprotein A-I, phospholipids, and cholesterol but are distinct by the variable presence of one or more representatives of at least 120 proteins and hundreds of lipid species. HDL are circulating multimolecular platforms that exert divergent functions such as reverse cholesterol transport, anti-inflammatory effects, anti-oxidative properties, immunomodulatory effects, endothelial protective effects, and improvement of endothelial function. These HDL functional properties are highly dependent on the proteome and lipidome of the particles and do not obligatorily correlate with HDL-cholesterol levels.

In Framingham Heart Study participants free of coronary heart disease at baseline, decreased HDL-cholesterol levels were independently associated with heart failure incidence after adjustment for interim myocardial infarction and clinical covariables [3]. Low HDL-cholesterol levels and low levels of apolipoprotein A-I, the principal apolipoprotein of HDL, carry an unfavourable prognosis in patients with heart failure independent of the aetiology [4,5]. Low HDL may be an integrated biomarker of adverse metabolic processes including abnormal metabolism of triglyceride rich lipoproteins, insulin resistance, and ongoing tissue inflammation. HDL dysfunction may contribute to both the occurrence and progression of heart failure. First, inflammation and heart failure are strongly interconnected. Secondly, heart failure is associated with systemic insulin resistance. Both inflammation and insulin resistance may lead to HDL dysfunction. HDL dysfunction and heart failure may mutually reinforce each other, *videlicet* a pattern of cyclic causality may be present.

Recent murine studies provide compelling evidence that HDL may exert direct effects on the myocardium that are completely independent of any effect on epicardial coronary arteries [6,7].

The HDL receptor SR-BI (scavenger receptor class B, type I) is a key regulator of lipoprotein metabolism and cholesterol homeostasis [6]. SR-BI binds HDL with high affinity and is expressed primarily in the liver and nonplacental steroidogenic tissues. Selective uptake of cholesterol by hepatic SR-BI and subsequent routing into bile is a major route for delivery of peripheral cholesterol to the liver for excretion in both mice and humans. *Scarb1*-deficient mice, lacking SR-BI protein expression, are characterized by increased plasma cholesterol comprising predominantly enlarged HDL enriched in free cholesterol and apolipoprotein E [6]. Lack of hepatic SR-BI activity results in dysfunctional HDL, characterized by a reduced capacity to promote cholesterol efflux, a decreased anti-oxidative potential resulting in increased oxidative stress, and impaired anti-inflammatory and pro-survival signalling. Transverse aortic constriction is a commonly used technique to induce pressure overload and induces HFrEF. Dysfunctional HDL in *Scarb1*^-/-^ mice leads to cardiac dysfunction both in the absence and in the presence of pressure overload and aggravates cardiac hypertrophy and adverse ventricular remodelling in mice with pressure overload [6]. In addition, heart failure was more pronounced in *Scarb1*^-/-^ mice with pressure overload as indicated by the more prominent increase of the lung weight compared to wild-type mice. These detrimental effects of SR-BI deficiency were nullified by hepatocyte-restricted SR-BI expression following SR-BI gene transfer, which restores HDL metabolism.

Improvement of HDL function following adeno-associated viral serotype 8-human apolipoprotein A-I gene transfer in C57BL/6J *low density lipoprotein receptor*^-/-^ mice enhanced cardiac function both in the absence and in the presence of pressure overload and counteracted cardiac hypertrophy and adverse ventricular remodelling in mice with pressure overload [7]. Heart failure following pressure overload was prevented by adeno-associated viral serotype 8-human apolipoprotein A-I gene transfer. Taken together, these findings on the effect of improved HDL function are the mirror image of the observations in *Scarb1*^-/-^ mice characterized by HDL dysfunction (Figure 1).

**Figure 1:**
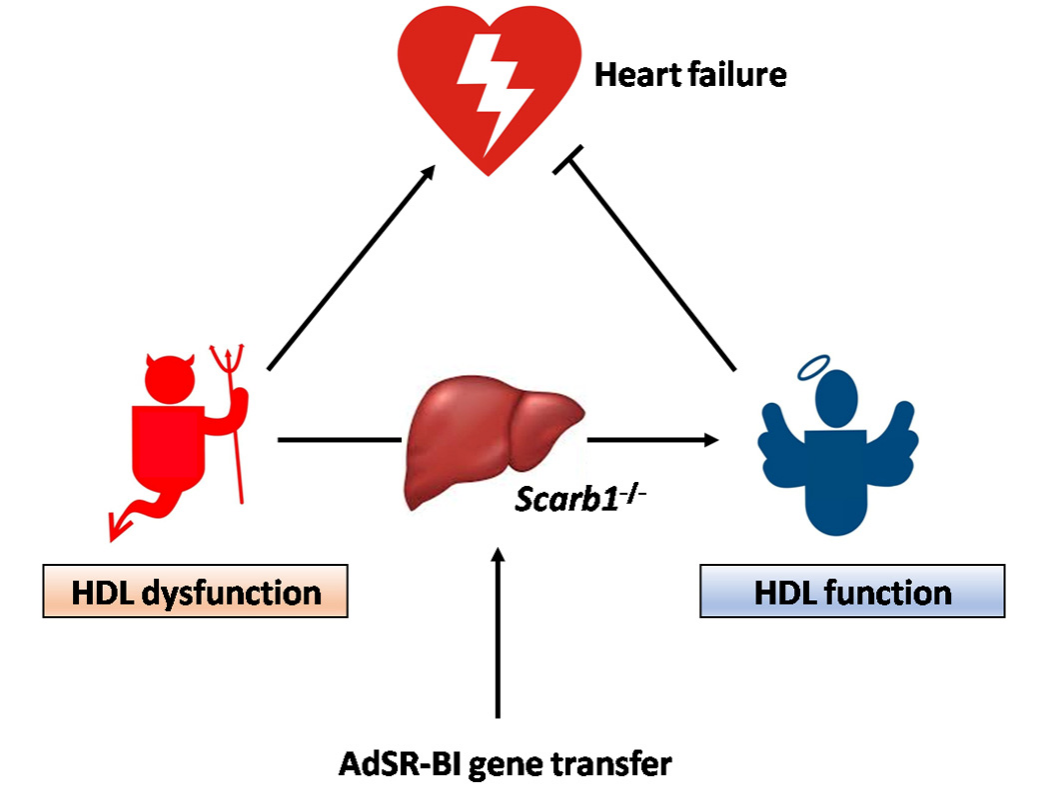
HDL dysfunction in *Scarb1*^-/-^ mice promotes heart failure whereas restoration of HDL function following AdSR-BI gene transfer inhibits heart failure.

Finally, intervention studies in mice with established heart failure have demonstrated that reconstituted HDL may reverse both HFrEF and HFpEF [2,8]. Specifically, administration of apolipoprotein A-I_Milano_/phospholipid complexes decreased left ventricular weight, increased myocardial capillary density, decreased interstitial and perivascular fibrosis, normalized systolic and diastolic function, and restored lung weight or improved exercise capacity. In all these studies, coronary atherosclerosis was documented to be absent.

In conclusion, HDL function appears to have a major impact on myocardial biology. Reconstituted HDL may emerge as a treatment modality for heart failure. Adeno-associated viral serotype 8-human apolipoprotein A-I gene transfer may have a potential for both prevention and treatment of heart failure.

## References

[1] Owan TE, et al. N Engl J Med. 2006; 355:251–59. 10.1056/NEJMoa052256 16855265

[2] Mishra M, et al. Int J Mol Sci. 2018; 19:3399. 10.3390/ijms19113399 30380754 PMC6274776

[3] Velagaleti RS, et al. Circulation. 2009; 120:2345–51. 10.1161/CIRCULATIONAHA.109.830984 19933936 PMC3600834

[4] Mehra MR, et al. J Heart Lung Transplant. 2009; 28:876–80. 10.1016/j.healun.2009.04.026 19716038

[5] Iwaoka M, et al. J Card Fail. 2007; 13:247–53. 10.1016/j.cardfail.2007.01.007 17517342

[6] Muthuramu I, et al. Arterioscler Thromb Vasc Biol. 2018; 38:2028–40. 10.1161/ATVBAHA.118.310946 29976771

[7] Amin R, et al. Int J Mol Sci. 2017; 18:2012. 10.3390/ijms18092012 28930153 PMC5618660

[8] Aboumsallem JP, et al. Br J Pharmacol. 2018; 175:4167–82. 10.1111/bph.14463 30079544 PMC6177616

